# Steal the rain: Interception loses and rainfall partitioning by a broad‐leaf and a fine‐leaf woody encroaching species in a southern African semi‐arid savanna

**DOI:** 10.1002/ece3.9868

**Published:** 2023-03-15

**Authors:** Felix V. Skhosana, Humbelani F. Thenga, Mohau J. Mateyisi, Graham von Maltitz, Guy F. Midgley, Nicola Stevens

**Affiliations:** ^1^ School of Climate Studies and Department of Botany and Zoology Stellenbosch University Matieland South Africa; ^2^ Council for Scientific and Industrial Research (CSIR) Pretoria South Africa; ^3^ Environmental Change Institute, School of Geography and the Environment University of Oxford Oxford UK

**Keywords:** functional traits, rainfall interception, stemflow, throughfall, woody plant encroachment

## Abstract

Woody plant encroachment (WPE) has been found to alter ecosystem functioning and services in savannas. In rain‐limited savannas, increasing woody cover can reduce streamflow and groundwater by altering evapotranspiration rates and rainfall partitioning, but the ecological relevance of this impact is not well known. This study quantified the altered partitioning of rainfall by two woody plant structural types (fine‐ and broad‐leaved trees) across a gradient of encroachment in a semi‐arid savanna in South Africa. Averaged across both plant functional types, loss of rainfall through canopy interception and subsequent evaporation roughly doubled (from 20.5% to 43.6% of total rainfall) with a roughly 13‐fold increase in woody cover (from 2.4 to 31.4 m^2^/ha tree basal cover). Spatial partitioning changes comprised fourfold increases in stemflow (from 0.8% to 3.9% of total rainfall) and a decline in throughfall proportion of about two‐fifths (from 80.2% to 47.3% of total rainfall). Changes in partitioning were dependent on plant functional type; rainfall interception by the fine‐leaved multi‐stemmed shrub *Dichrostachys cinerea* was almost double that of the broad‐leaved tree *Terminalia sericea* at the highest levels of woody encroachment (i.e., 49.7% vs. 29.1% of total rainfall intercepted at tree basal area of 31.4 m^2^/ha)*.* Partitioning was also dependent on rainfall characteristics, with the proportion of rainfall intercepted inversely related to rainfall event size and intensity. Therefore, increasing tree cover in African grassy ecosystems reduces the amount of canopy throughfall, especially beneath canopies of fine‐leaved species in smaller rainfall events. Rainfall interception traits may thus confer a selective advantage, especially for fine‐leaved woody plant species in semi‐arid savannas.

## Graphical abstract

This study quantified the altered partitioning of rainfall by two woody plant structural types (fine‐ and broad‐leaved trees) across a gradient of encroachment in a semi‐arid savanna in South Africa. At the highest woody cover, rainfall loss through canopy interception by a fine‐leaved shrub *Dichrostachys cinerea* almost doubled that of a broad‐leaved tree *Terminalia sericea*. Rainfall partitioning was also dependent on rainfall characteristics, with the proportion of rainfall intercepted inversely related to rainfall event size and intensity.

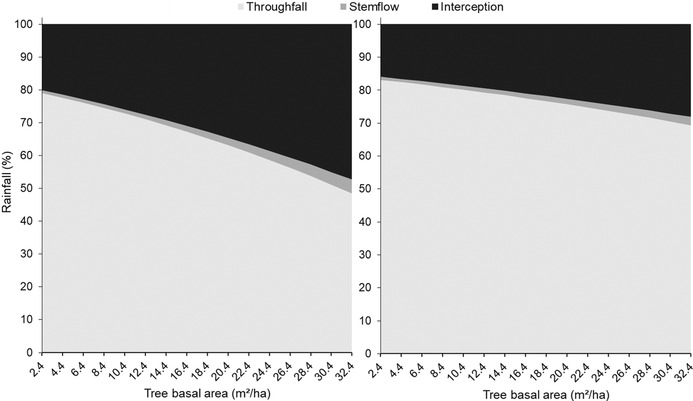

## 1 INTRODUCTION

Savannas are characterized by discontinuous tree canopy in a continuous grass layer and cover about 20% of the Earth's and over 50% of Africa's land surface area (Scholes & Archer, 1997; Wang et al., 2009). Savannas are experiencing significant increases in woody plant encroachment—where indigenous woody cover is increasing (Stevens et al., 2017; Venter et al., 2018). The continued increase in woody cover within non‐forested systems threatens biodiversity and alters the structure and function of savannas and the ecosystem services they provide (de Klerk, 2004; Eldridge et al., 2011; Meik et al., 2002). The increased woody cover shades out the grassy layer, reducing grass biomass and hence forage availability (Belay & Moe, 2015; Charles‐Dominique et al., 2018; Randle et al., 2018).

In water‐limited systems, woody encroachment impacts the ecosystem hydrology by reducing above‐ and below‐ground water availability through increasing evapotranspiration (Archer, 2010; Grygoruk et al., 2014; Scott et al., 2006; Wang et al., 2018) when deeper‐rooted trees access deeper soil layers resulting in drying the soil during transpiration (Scott et al., 2006). High levels of encroachment also cause water loss via evaporation through canopy interception, where rainfall landing on the leaves of a tree canopy remains on the leaves and gets evaporated (Dohnal et al., 2014; Honda & Durigan, 2016). The partition of rainfall into interception, throughfall, and stemflow has been extensively quantified and documented in forest systems, however, similar studies are still lacking in savanna systems, especially in Africa (Magliano et al., 2019). In forests, canopy interception losses have been shown to reduce as much as 10%–50% of rainfall reaching the ground, with this amount varying with forest characteristics and climate (Carlyle‐Moses & Gash, 2011; Roth et al., 2007). Similarly, in arid shrublands, as much as 9%–25% of rainfall can be lost to interception by vegetation canopies (Magliano et al., 2019; Návar et al., 1999; Zhang et al., 2015, 2017). Yet, we cannot generalize findings from other biomes to savanna ecosystems due to differences in dominant plant functional types and the differences in the stem and canopy architectures of the trees. Forest trees, for example, are taller with wider canopy diameter for a given basal area and have higher specific leaf area (Archibald & Bond, 2003; Ratnam et al., 2011). This, therefore, remains a significant gap in savannas, especially African savannas, as it is not well documented how much canopy interception occurs generally and or how this component of the water cycle shifts with woody encroachment (Honda & Durigan, 2016; Savenije, 2004).

The relationship between canopy interception and encroachment might also be altered by the type of rainfall. A small and less intense rainfall event is more likely to have more rain intercepted by the canopy and evaporated back into the atmosphere. Rainfall in semi‐arid savannas is characterized by a few large rainfall events and several small rainfall events while mesic savannas are characterized by a few small rainfall events but many large rainfall events (Pilgrim et al., 1988). If this holds true, encroached arid and semi‐arid savannas that receive relatively small rainfall events will experience more significant losses of rainfall through interception.

The architecture of trees is also likely to be a crucial factor that shapes how much rainfall is lost to interception. In Southern African semi‐arid savanna systems, where woody encroachment is prevalent (Stevens et al., 2017), encroachment is dominated by two plant functional types; thorny fine‐leaved leguminous shrubs such as *Dichrostachys cinerea*, *Vachellia*, and *Senegalia* species, and by broad‐leaved trees such as *Terminalia sericea* and *Combretum* species (Ben‐Shahar, 1992; Roques et al., 2001). Savanna broad‐leaf trees are shorter than forest trees but are taller and have wider canopies than fine‐leaved species (Moncrieff et al., 2014). Fine‐leaved species on the other hand are relatively shorter and shrubby with wide and round canopies that are much closer to the ground (Moncrieff et al., 2014). Therefore, the architectural differences between these functional types (Randle et al., 2018) may result in differences in rainfall partitioning, as previous studies show architectural differences lead to distinct ecosystem impacts when their cover increases (Belay & Moe, 2015; Moncrieff et al., 2014; Osborne et al., 2018; Randle et al., 2018; Zizka et al., 2014).

To address this gap, we examined how the partitioning of rainfall changed across the gradient of woody plant encroachment in a semi‐arid African savanna. We investigated how this relationship was modified by (i) plant functional types (fine‐leaf *D. cinerea* vs. broad‐leaf *T. sericea*) and (ii) rainfall characteristics (size and intensity).

## 2 MATERIALS AND METHODS

### 2.1 Study area

This study was located at Wits Rural Facility (WRF; 24°31′S; 31°06′ E), a 350‐ha research station of the University of the Witwatersrand, in the Lowveld savanna within the Limpopo Province of South Africa. This semi‐arid savanna receives a mean annual rainfall of ~650 mm, mostly concentrated in the summer season (October to April), mainly in the form of conventional thundershowers (Kaschula et al., 2005; Moyo, 2013; Neke et al., 2006). Periodic droughts occur ~ once every 4 years (Neke, 2005). The mean annual temperature is 22°C (Neke et al., 2006; Shackleton, 1993). The most common soil types in the region are shallow sandy nutrient‐poor lithosols derived from granite gneiss (Kaschula et al., 2005; Neke et al., 2006; Shackleton, 2001).

The vegetation type is classified as Granite Lowveld savanna and is dominated by tree species in the Combretaceae (e.g., *Terminalia sericea* and *Combretum* species) and in the Fabaeae (e.g., *Dichrostachys cinerea*, *Vachellia*, and *Senegalia* species) families (Neke et al., 2006; Shackleton, 1993, 2001). The dominant grasses in this savanna are *Panicum maximum*, *Hyperthelia dissoluta*, and *Heteropogon contortus* (Shackleton, 1993).

### 2.2 Sampling procedure

**FIGURE 1:**
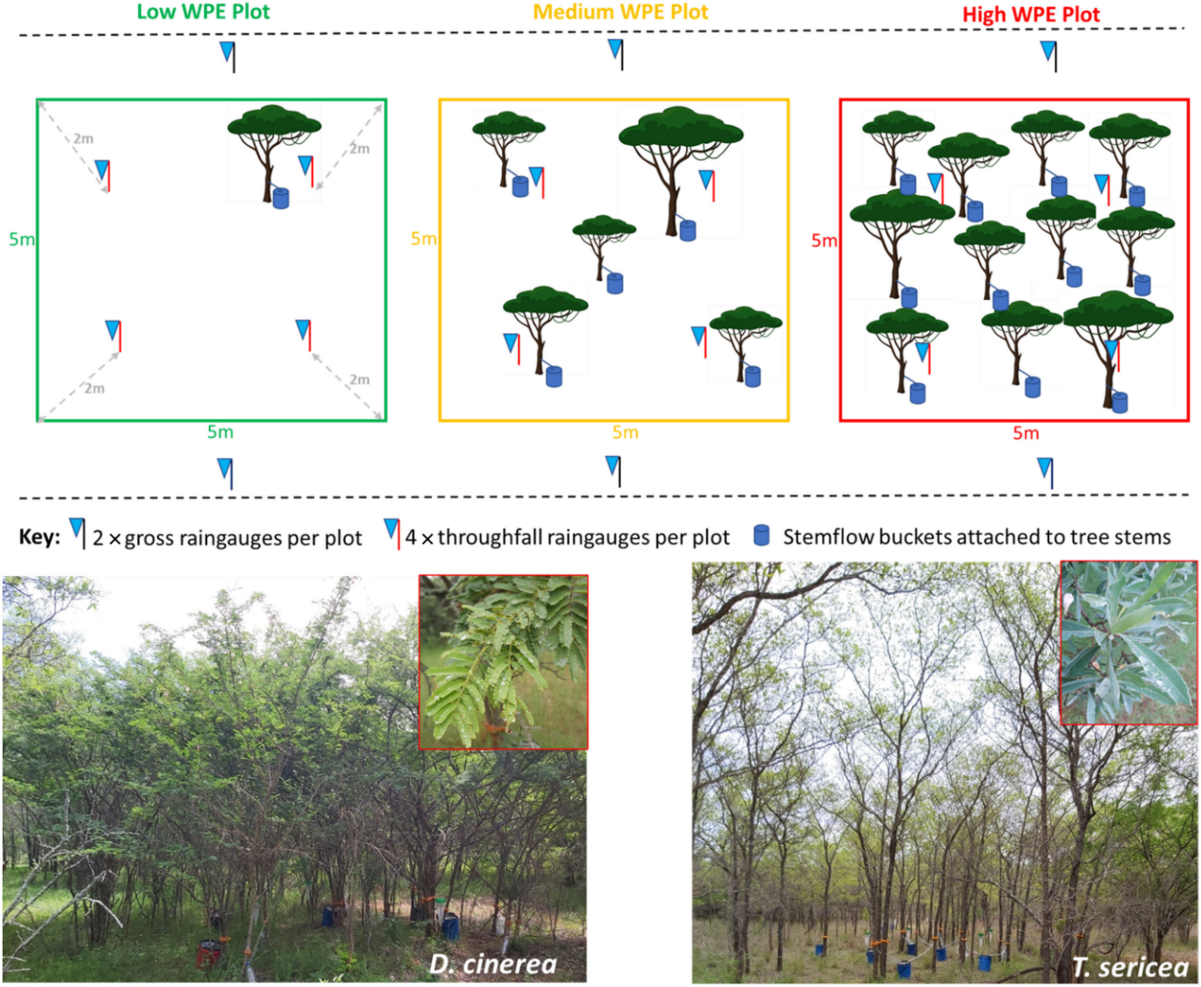
The experimental design to measure gross precipitation (2 × rain gauges in the open spaces outside each plot), throughfall (4 × rain gauges inside each plot), and stemflow (collected using buckets attached to stems) across a gradient of woody encroachment (low, medium, and high) for *Dichrostachys cinerea* and *Terminalia sericea*. The two pictures represent a highly encroached plot for each of the two species.

To investigate the influence of woody plant encroachment on rainfall partitioning, we established three 5 × 5 m plots per species across a gradient of woody encroachment with each plot replicated three times. This was repeated for two species (*Dichrostachys cinerea* and *Terminalia sericea*) (Figure 1, and see Randle et al., 2018, for a detailed description of these two species). Three levels of encroachment were selected: low, medium, and high. The levels of encroachment were initially characterized by stem counts in the 5 × 5 m plots. Low encroachment plots were represented by 1–2 individual trees, medium by 5–6 individual trees, and high by more than 10 individual trees (Figure 1). Between the two species, *D. cinerea* has lots of stems and *T. sericea* does not, therefore, to standardize, stems further than 30 cm apart were counted as separate trees. To avoid influence by other tree species, we only selected plots that exclusively contained the study species of interest (*D. cinerea* and *T. sericea*). Every treatment (species × encroachment level) was replicated three times with three 25 m^2^ plots located between 0.5 and 2 km from each other. No plot was located more than 2 km apart to ensure similar rainfall events and soil conditions. For multi‐stemmed trees, we defined individual trees as single stems coming from the ground more than 30 cm apart.

#### 2.2.1 Measurements of vegetation characteristics

At the beginning of the study, each 25 m^2^ plot was surveyed to characterize vegetation. For each tree, we measured height, stem diameter (at 0.5 m height), stem density (number of stems per plot), and canopy cover (the percentage of ground area shaded by overhead foliage). The canopy cover was quantified using a Cl 110 Plant Canopy Imager (https://cid‐inc.com/) in the form of leaf area index (LAI). Measurements were taken by extending the arm inside the plot every 2.5 m along the perimeter of the plot, adding to a total of 8 points measured inside a 5 m × 5 m plot. Due to the wide field of view of the fish‐eye lens, eight measurements covered the entire area of the plot. Canopy cover was done during summer for all plots to reduce seasonal bias. Dense stands (highly encroached) of *D. cinerea* had multiple thin stems with a high canopy cover (Table S1; Figure S1), and dense stands (highly encroached) of *T. sericea* on the other hand were characterized by few, thick stems with a lower LAI than *D. cinerea* (Table S1; Figure S1). Tree basal area (TBA) was calculated for each plot using the formula:

(1) $$\mathrm{TBA}\;\left(m^{2}{\mathrm{ha}}^{-1}\right)=\sum \left\{\pi {\left(\;\frac{\mathrm{DBH}}{2}\;\right)}^{2}\right\}/\mathrm{ha}$$

where *DBH* is the diameter (m^2^) of each stem within the plot at breast height and ha is the area of the plot.

#### 2.2.2 Measurements of rainfall partitioning and rainfall characteristics

The quantification of gross precipitation and rainfall partitioning into stemflow, throughfall, and interception was done for each plot (9 plots per species) during the rainy season between November and March of the years 2019–2020 and 2020–2021 when the trees were in full canopy. A rainfall event was defined as a measurable amount of rainfall separated from the previous or the next rainfall input by the time required for the foliage and trunks to dry (Krämer & Hölscher, 2009). In our study site, the foliage and trunk dried between 3 and 5 h, so each rainfall event was considered a separate event after 5 h had passed. In each plot, gross precipitation (the total amount of rain that falls on the ground in the open) was quantified using two rain gauges installed in open spaces (Figure 1). Each rain gauge had a catchment area of 133 cm^2^ and was installed at a height of 0.5 m aboveground. The amount of water collected in the rain gauge was measured using a measuring cylinder and then emptied after each rainfall event. The rain gauges for measuring throughfall and the buckets for measuring stemflow were coated with oil to avoid evaporation of the collected water. The duration of each rainfall event was obtained from a tipping bucket rain gauge installed in between the plots to be able to calculate rainfall intensity later. Regular inspections and maintenance work was done between rainfall events to ensure that all rain gauges were in place and that no collars were broken.

Rainfall data were partitioned into throughfall, stemflow, and interception. To get a value equivalent to the standard mm of rainfall, each rainfall partition was calculated as per unit area (Lm^− 2^). The average gross precipitation from the two rain gauges installed around each plot (Figure 1) was calculated as:

(2) $$\text{Gross rainfall}\;\left({\mathrm{Lm}}^{-2}\right)=\frac{\text{mean rainfall in the open}}{\text{catchment area of rain gauge}}$$

To measure throughfall in each plot, four rain gauges were installed beneath tree canopies (Figure 1). Throughfall rain gauges were placed at a 2 m distance from the nearest plot corner (Figure 1), and they were placed so they did not touch any stems or branches to avoid heavy run‐off from the trunks (Rutter, 1963). The rain gauges' positions within the respective plots were kept fixed to capture the intensities of different rainfall events. Rainfall in each rain gauge was measured after each rainfall event using measuring cylinders. Plot‐level throughfall and throughfall proportion were calculated as:

(3) $$\text{Throughfall}\;\left({\mathrm{Lm}}^{-2}\right)=\frac{\text{mean plot rainfall}}{\text{catchment area of rain gauge}}$$

(4) $$\text{Proportion throughfall}\;\left(P_{\mathrm{tf}}\right)=\frac{\text{Throughfall}}{\text{Gross rainfall}}$$

Stemflow was quantified using collars (made of polyurethane foam) with an internal diameter of approximately 5 cm which were attached around every stem within the plot at a height of ~0.5 m from the ground (Honda & Durigan, 2016; Krämer & Hölscher, 2009). The collars were sealed with silicone sealant ensuring that water leakages are avoided. Flexible tubes channeled the water into containers (Figure 1). The total stemflow and proportion of stemflow measured per plot in each rainfall event were calculated as:

(5) $$\text{Stemflow}\;\left({\mathrm{Lm}}^{-2}\right)=\frac{\text{total plot stemflow}}{\text{area of the plot}}$$

(6) $$\text{Proportion stemflow}\;\left(P_{\mathrm{sf}}\right)=\frac{\text{Stemflow}}{\text{Gross rainfall}}$$

The total amount of rainfall (net) and the proportion of rainfall reaching the ground in the plots were calculated as:

(7) $$\mathrm{Net}\;\text{rainfall}\;\left({\mathrm{Lm}}^{-2}\right)=\text{Throughfall}+\text{Stemflow}$$

(8) $$\text{Proportion}\;\mathrm{net}\;\left(P_{\mathrm{net}}\right)=P_{\mathrm{tf}}+P_{\mathrm{sf}}$$

Canopy interception and the proportion of interception for each rainfall event in each plot were calculated as:

(9) $$\text{Interception}\;\left({\mathrm{Lm}}^{-2}\right)=\text{Gross rainfall}-\mathrm{Net}\;\text{rainfall}$$

(10) $$\text{Proportion interception}\;\left(P_{\mathrm{int}}\right)=1-P_{\mathrm{net}}$$

### 2.3 Characteristics of rainfall at the study site

**FIGURE 2:**
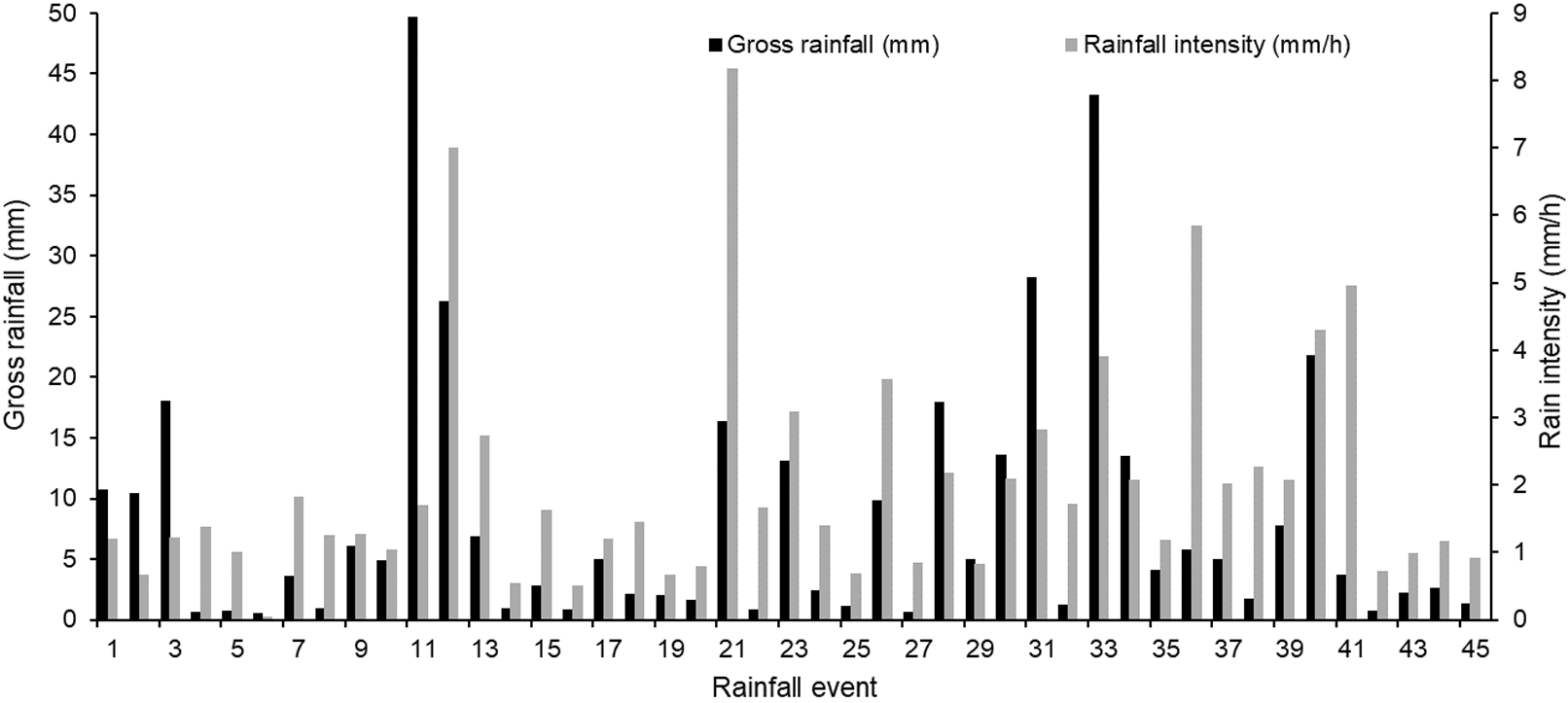
Gross rainfall and rainfall intensity for 45 rainfall events in the study area during the growing seasons from 2019 to 2021.

A total of 45 rainfall events were recorded during the two growing seasons between 2019 and 2021. Mean annual rainfall was 389.8 mm and rain events ranged from 0.5 to 49.7 mm with an average of 8.4 mm across rainfall events (Figure 2). Rainfall intensity (gross × duration) ranged from 0.1 to 8.2 mm/h with an overall mean of 2 mm/h per rainfall event during the sampling period (Figure 2).

Rainfall events were grouped linearly into three classes of 0–10 mm representing low, 10–20 mm medium, and >20 mm representing high rainfall size based on the classification of indices of climate extremes by the Expert Team on Climate Change Detection and Indices (ETCCDI; Klein Tank et al., 2009). Rainfall intensity was also linearly categorized into two groups of light (trace–2.5 mm/h) and moderate (2.6–7.5 mm/h) rainfall as guided by the standard classifications by the American Meteorological Organization (AMO) (Barthiban et al., 2012). The 45 rainfall events were clustered into three rainfall classes: 31 (68.9%) fell into the 0–10 mm (low) rainfall class (Table 1); 9 events (20.0%) and 5 events (11.1%) in the 10–20 mm (medium); and >20 mm (large) rainfall classes, respectively (Table 1). The large rainfall class contributed 169.3 mm which is 43.4% of the total rainfall recorded, followed by the medium class with 124.3 mm (31.9%), and lastly, the low class with 96.2 mm (24.7%) (Table 1). The large class had the highest rainfall intensity (3.9 mm/h) (Table 1). It was followed by the medium with 2.7 mm/h and the low rainfall class with 1.6 mm/h rainfall intensity (Table 1). Even though all plots were located within a 2 km radius, a few rainfall events showed local spatial variation. Therefore, to reduce the effect of this on the analysis, we selected 40 of the 45 events with a coefficient of variation of less than 15%, as done by Honda and Durigan (2016).

**TABLE 1:** Characteristics of rainfall within the study area during the sampling period.

Rainfall class (mm)	Rain frequency	Frequency (%)	Rain (mm)	Rain (%)	Rain intensity mean (mm/h)	Rain intensity range (mm/h)
0–10 mm (low)	32	71.1	96.5	25.4	1.6	0.06–5.85
10–20 mm (medium)	8	17.8	114.0	30.0	2.6	0.68–8.18
>20 mm (large)	5	11.1	169.3	44.6	4.0	1.7–7.00

### 2.4 Data analysis

All data analyses were performed in R v. 4.1.3 (R Development Core Team, 2021). Correlations were run between woody cover variables (TBA, LAI, and stem density) to establish if there were any associations between the variables thereby informing which variable could be used interchangeably in representing the effect of WPE on rainfall partitioning. The tree basal area (TBA) positively correlated (*r* ^2^ > .5) with stem density and LAI (Figure S1). To maintain consistency with other previous similar studies (such as Honda & Durigan, 2016), TBA was chosen as a proxy for woody cover among the three measures. It ranged from 2.4 to 31.4 m^2^/ha at plot level.

To determine the distributions of the three rainfall partitions (proportion throughfall, stemflow, and interception), we used the **“*gamlss.dist*”** R package which contains about 59 types of distributions grouped into four sets (“*realAll*,” “*realline*,” “*realplus*,” and “*real0to1*”), which can be used for modeling a continuous response variable using generalized additive models for location scale and shape (GAMLSS) (Rigby & Stasinopoulos, 2005). The function *“fitDist”* was used to fit “*realAll”* (all the 51 *gamlss.family* continuous distributions defined on the real line (i.e., “r*ealline”* and the real positive line, i.e., “*realplus”*)) and “*real0to1*” (all eight *gamlss.family* continuous distributions from 0 to 1) on each of the rainfall partitions. The final marginal distributions were ranked by their generalized Akaike information criterion (GAIC) (Rigby & Stasinopoulos, 2005). When comparing the fitted distributions, we found that a beta (“BE”) distribution was consistently in the top 2 of the distributions that best represented the data (number 1 for Throughfall and number 2 for Stemflow and Interception) together with other distribution types such as the *Skewed Normal type 2* (*“SN2”*), the *Generalized Gamma* (*“GG”*), and the Box–Cox Power Exponential “BCPEo” (See the Figure S2a for the BE fitting). We, therefore, ran Generalized mixed effects models using the “***gamlss***” R package with the top 3 distributions for each of the rainfall partitions, proportion throughfall (*P*_tf_), stemflow (*P*_sf_), and interception (*P*_int_) in response to TBA, species, rainfall class, and rainfall intensity class. To account for variability across sites, we used “site” as a random effect. The general setup of the functions for *P*_tf_, *P*_sf_, and *P*_int_ as response variables to treatments (TBA, species, rainfall class, and rainfall intensity) as fixed effects and site as a random effect was as follows:

(11) $$\text{gamlss}\left(y\sim \text{mixed effects}+\text{random effects},\text{data},\text{family}\right)$$

To determine the best model that represented each of the rainfall partitions, we compared the models using their AIC scores and the distributions of the model residuals using Q–Q plots. The best‐fitting model is represented by having the lowest AIC score (Wagenmakers et al., 2004). Again, we found that the beta (*BE*)‐based models consistently fitted the data distribution in comparison with other models, being ranked first for *P*_tf_, second for *P*_sf_ after the *generalized beta type 1* (*GB1*), and second for *P*_int_ after *generalized gamma* (*GG*). To maintain consistency and avoid bias, we, therefore, used the beta distribution throughout (see the Figure S2b for the fitting of the residuals for *BE* models). The significance level for all tests was assumed at *p* < .05. To predict the response variables from the model outputs, the “*ggpredict*” function within the “***ggeffects***” package was used. For simplicity, and for creating graphs, the predicted proportions (*P*_tf_, *P*_sf_, and *P*_int_) were transformed into percentages (*P*_tf_%, *P*_sf_%, and *P*_int_%) of the gross by multiplying by 100.

## 3 RESULTS

**FIGURE 3:**
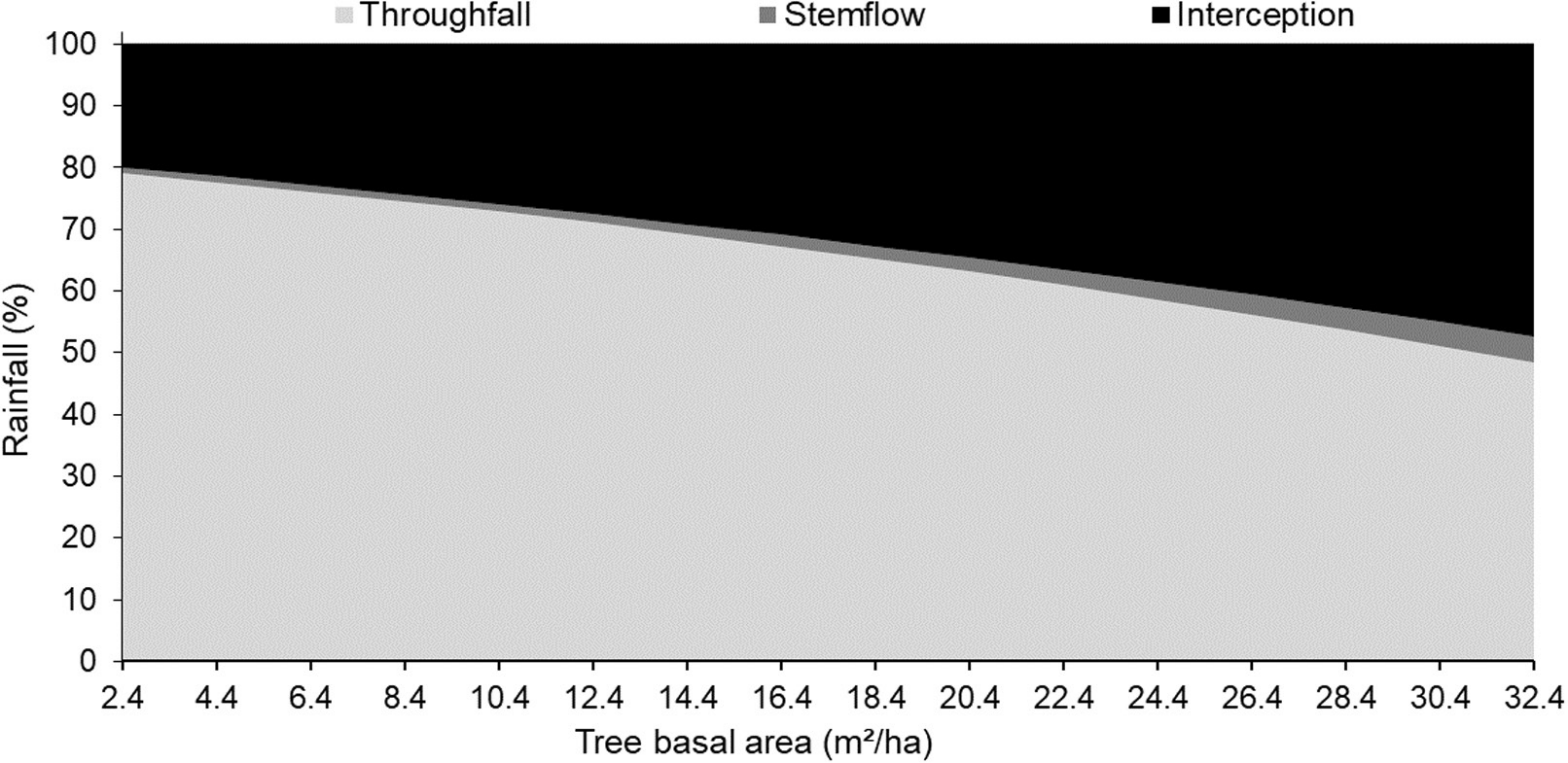
The accumulation of rainfall partitioning into interception, throughfall, and stemflow in response to an increase in tree basal area (TBA) as per the predictions of the beta regression model.

Woody encroachment changes rainfall partitioning by increasing interception and stemflow. The amount of rainfall intercepted by the tree canopies increased across the gradient (TBA: 2.4–31.4m^2^/ha) of woody encroachment. At the lowest woody cover, 20.5% of the rainfall was intercepted while at the highest woody cover 43.6% of the rainfall was intercepted (Figure 3). As tree basal area increased the amount of rainfall reaching the ground through stemflow increased from 0.8% at the lowest TBA to 3.9% at the highest TBA (Table S2; Figure 3). This, however, did not offset the increased losses from interception as throughfall declined from 80.2% at the lowest TBA to 47.3% at the highest TBA resulting in an overall 33.6% decrease in the amount of rainfall reaching the ground with woody encroachment (Table S2; Figure 3).

**FIGURE 4:**
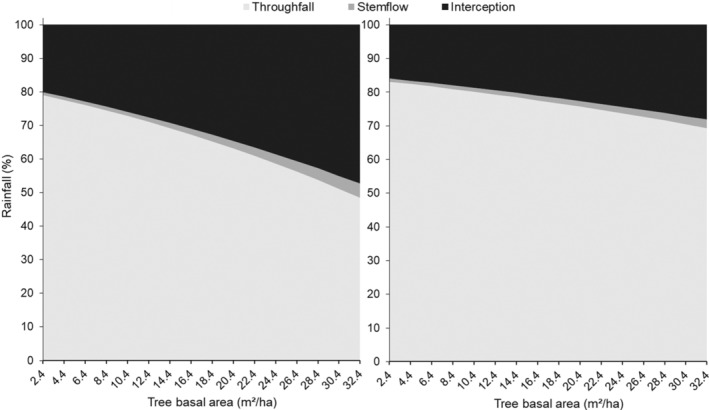
The accumulation of rainfall partitioning into interception, throughfall, and stemflow in response to an increase in tree basal area (TBA) for *Dichrostachys cinerea* and *Terminalia sericea* as per the predictions of the beta regression model.

This interaction between woody encroachment and rainfall partitioning was further modified by plant functional types. Rainfall partitioning significantly differed between the two species (*p* < .05) (Table S2) and on average interception loss was significantly higher (*p* < .001) for fine‐leaved multi‐stemmed shrub *D. cinerea* (30.0%) when compared to the broad‐leaved tree *T. sericea* (20.7%). *D. cinerea* also had significantly (*p* < .001) lower throughfall (67.1%) relative to *T. sericea* (78.0%) while stemflow was significantly (*p* < .001) higher in *D. cinerea* (1.7%) compared to *T. sericea* (1.4%). Moreover, interception and stemflow continued to increase across the gradient of woody cover and throughfall continued to decline in both species. At the lowest woody cover, 20% of the rainfall was lost under *D. cinerea* compared to 16% under *T. sericea*, and as TBA increased to the highest, more interception losses occurred for *D. cinerea* (49.7%) relative to *T. sericea* (29.1%; Figure 4). Similarly, as the number of stems increased, stemflow increased from 0.9% to 4.7% for *D. cinerea* and 0.9% to 2.7% for *T. sericea*, and throughfall decreased from 79.1% to 45.6% for *D. cinerea* and 83.1% to 68.1% for *T. sericea* from lowest (TBA = 2.4 m^2^/ha) to highest (31.4 m^2^/ha) woody cover (Figure 4).

**FIGURE 5:**
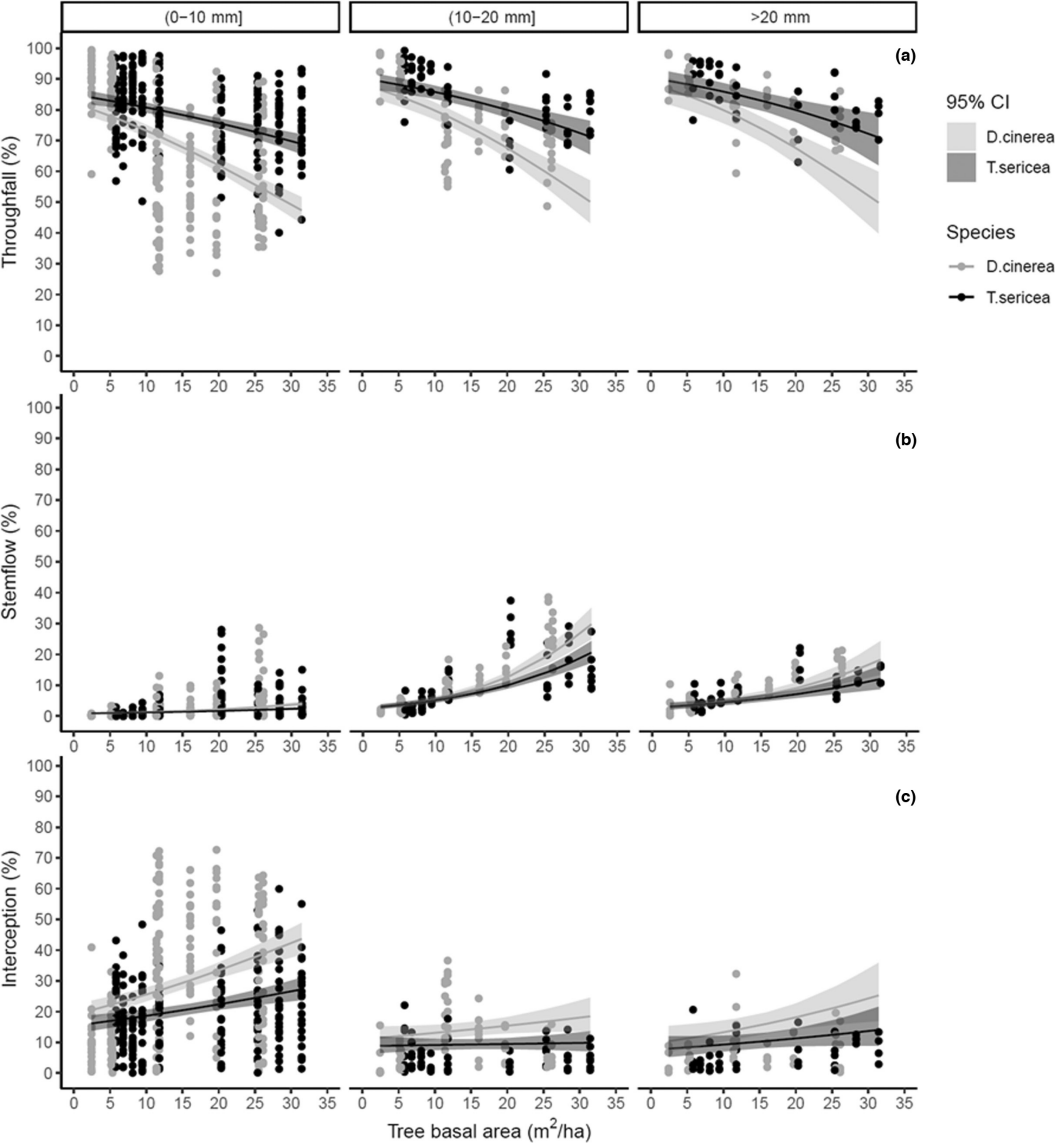
Rainfall partitioning into interception (a), stemflow (b), and throughfall (c) across a gradient of TBA by the two species and rainfall classes. The black curves represent the predicted values of each partition, and the upper and lower bands represent 95% confidence intervals (95% CI). These curves with 95% CI are fitted on the actual dataset (black dots on the scatter plots).

The relationship between woody encroachment and rainfall partitioning was further influenced by rainfall size and intensity (Table S2). The amount of rainfall event had a negative effect on the proportion of rainfall lost through interception, where significantly less rainfall was lost to interception and more was gained from increased stemflow during large rainfall events (Figure 5). At the highest level of woody encroachment, interception loss decreased from 43.7% for *D. cinerea* and 24.4 for *T. sericea* when rainfall events were small (0–10 mm) to 23.4% and 11.3%, respectively, when the events were large (>20 mm) (Figure 5). As a result, less rain, 51.2% for *D. cinerea* and 71.3% for *T. sericea*, reached the ground during low rainfall events (0–10 mm) compared to 68.1% for the former and 83% for the latter at high rainfall sizes (>20 mm) within the dense plots (31.4 m^2^/ha; Figure 5).

**FIGURE 6:**
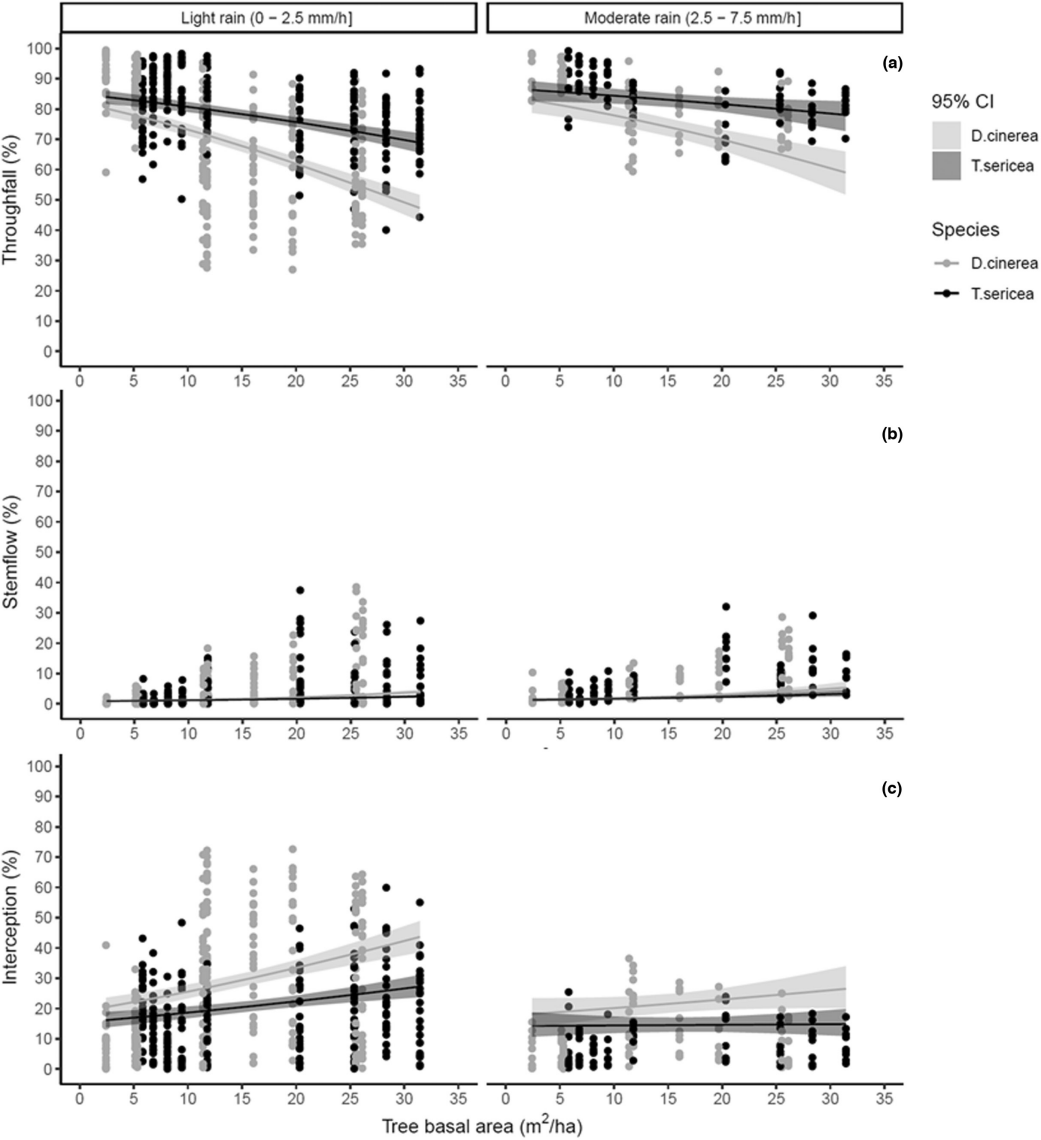
Rainfall partitioning into interception (a), throughfall (b), and stemflow (c) across a gradient of TBA by the two species and rainfall intensity. The black curves are the predicted values of each partition, and the upper and lower bands represent 95% confidence intervals (95% CI) from the beta regression model. These curves with 95% CI are fitted on the actual dataset (black dots on the scatter plots).

Similarly, rainfall intensity also significantly altered interception losses (*p* = .03; Table S2) where the percentage of rainfall reaching the ground increased as rainfall intensity increased due to a decline in rainfall interception (Table S2; Figures 5 and 6). At the highest woody cover, low rainfall intensities resulted in higher interception losses of 43.6 for *D. cinerea* and 27.2% for *T. sericea*, whereas high intensities resulted in lower (26.5% and 14.8%, respectively) interception losses (Figure 6). Ultimately, less rainfall reached the ground (51.2% for *D. cinerea* and 71.3% for *T. sericea*) during less intense rainfall events (0–2.5 mm/h), in comparison to 64.3% and 81.3% for the respective species during more intense events (2.5–7.5 mm/h; Figure 6).

## 4 DISCUSSION AND CONCLUSION

Woody encroachment significantly changes rainfall partitioning by increasing interception loss and stemflow and reducing throughfall. Based on this study, this results in approximately 44% of rainfall captured by woody canopies and evaporated back into the atmosphere at the highest levels of encroachment. This relationship is modified by the type of encroaching plants and the characteristics of the rainfall regime (size and intensity). About 9% less rain enters the soil when a system is encroached by a fine‐leaved multi‐stemmed shrub such as *D. cinerea* rather than a broad‐leaved tree‐like *T. sericea*. Notably, this relationship is also impacted by rainfall characteristics and encroached areas where rainfall intensity and size are frequently large, experience less loss to interception than areas where the rainfall regime is characterized by frequent small rainfall events.

We showed that rainfall partitioning changed across a gradient of increasing tree basal area where the increased cover increased interception and stemflow and decreased throughfall. A study in a mesic savanna in Brazil also demonstrated that an increase in interception and stemflow and a decline in throughfall occurred as woody cover increased over an encroachment gradient (Honda & Durigan, 2016). The increase in canopy cover results in fewer gaps within and between tree canopies and increases the capacity for canopies to hold more water. The particular effectiveness of shrubs in channeling rainfall has also been shown where increases in stemflow occurred with an increase in the number of stems per unit area and a lower angle of stem insertion as seen in shrubs (Levia & Frost, 2003; Zhang et al., 2017). Moreover, the multi‐stems of a shrub such as *D. cinerea* are vertically oriented, somewhat like an “inverted cone,” and likely making them more efficient in funneling stemflow to the stem bases. This is in contrast to the single‐stemmed morphology of taller trees, with branches ramifying in different directions from a certain height (Li et al., 2008; Martinez‐Meza & Whitford, 1996; Zhang et al., 2015).

The sensitivity of rainfall partitioning to plant functional type is an essential element to understand when modeling the impacts of encroachment on rainfall. The difference in rainfall partitioning is likely driven by species‐specific characteristics—from growth form, canopy architecture, stem orientation or angle, branching, and leaf traits such as shape, size, and angle (Crockford & Richardson, 2000; Levia & Frost, 2003; Pérez‐Suárez et al., 2014). There is evidence that leaves that are held vertically allow greater light penetration (Falster & Westoby, 2003) and likewise more rain – but also that highly branched canopies result in higher interception (Li et al., 2016; Pflug et al., 2021). The position of leaves on the shoot – clustered at the tips or distributed along the stem – can also impact net throughfall. *T. sericea* is probably on the extreme end of the throughfall spectrum, having its broad leaves clustered in groups at the branch tips, which are also generally held more vertically, and with a fairly tall unbranched main stem which could tend to increase throughfall. By contrast, *D. cinerea* has a highly branched stem (multi‐stem), with more lateral branches and leaves distributed throughout the branch length (Figure 1). The canopy is spheroid and deep with a relatively small crown‐base height compared to *T. sericea*, therefore, adding more water‐holding capacity (Belay & Moe, 2015; Martens et al., 2000; Randle et al., 2018). In addition, bipinnate leaves (fine leaves) have a high surface area‐to‐volume ratio allowing more surface area to retain water to evaporate back into the atmosphere (as shown in Figure 1). Consistent with this, Khan (1999) reported a higher interception loss (21.77% vs. 12.72%) by a fine‐leaf *Vachellia tortilis* when compared to a broad‐leaf *Colophospermum mopane* in an arid zone in India.

The pattern of shifting rainfall partitioning across a woody gradient is not only altered by the dominant woody encroaching species but also depends on the characteristics of the rainfall regime where small and low‐intensity rainfall events resulted in the highest rate of interception. With large rainfall size and intensity, tree canopies and stems quickly become saturated and the proportion of net rainfall increases (Carlyle‐Moses, 2004; Pérez‐Suárez et al., 2014; Zhang et al., 2015). Carlyle‐Moses (2004) showed a similar pattern in a semi‐arid system in northeast Mexico where there was an increase in throughfall with an increase in rainfall size until the canopy reached saturation at 20 mm. A similar relationship with rainfall regime has been documented across regions and biomes where a decline in relative interception loss due to canopy saturation occurs across multiple species (Owens et al., 2006; Zhang et al., 2015). Another explanation that is beyond the scope of this study could be linked to an increase in wind speed during stormy high‐intensity rainfall events where windy conditions reduce the size of water captured by tree canopies. This can also explain the observed high variability in the observed data, especially with *D. cinerea*, a shrub with relatively thinner stems that can be easily shaken by the wind.

In this study, we note that woody encroachment by *D. cinerea* and *T. sericea* results in less rainfall reaching the ground, especially at lower rainfall sizes and intensities. This is likely to be a generic result of encroaching species with comparable stem and leaf traits and canopy architecture. Differences emanating from tree branching ratios, length, branch angle, and leaf characteristics could lead to significant differences in the proportions of rainfall partitioning. When comparing the two plant species, we conclude that the interception by fine‐leaved *D. cinerea* is more pronounced than the broad‐leaved *T. sericea*. Debushing programs that target the proliferation of encroaching species in arid regions should therefore prioritize controlling fine‐leaved encroachers for optimum impact. This has important implications for conservation and management not only in savanna systems but also in all open grasslands that are threatened by woody encroachment. A reduction of ~10% in water availability is significant, especially when viewed in light of future changes in rainfall (Al‐Ansari et al., 2014; Milly et al., 2005). Reducing the size of water entering the ground will also have multiple interactive effects on other ecosystem services. For example, less water will result in less grass growth, which is already increasingly limited by increased shading driven by woody encroachment (Belay & Moe, 2015; Randle et al., 2018). Alternatively, the decrease in available water could make these encroaching trees more susceptible to drought and heat‐related death (Case et al., 2019).

These results highlight an important consequence of increasing tree cover in grassy ecosystems and provide a valuable counter to the argument for increasing tree cover in grassy ecosystems as a tool to increase carbon sequestration (Bastin et al., 2019; Bond et al., 2019; Tear et al., 2021). We show that aside from reducing carbon grass biomass and biodiversity, high tree cover will also significantly reduce effective rainfall, especially in these semi‐arid systems (Andersen & Steidl, 2019; Randle et al., 2018). In addition to the established differences in canopy interception with plant functional types, there is a need for exploring the role of individual tree characteristics such as canopy architecture and leaf traits in providing a selective advantage in arid and semi‐arid savannas. This perspective could be a valuable line of inquiry in further studies on the drivers and consequences of woody plant encroachment.

## AUTHOR CONTRIBUTIONS

**Felix V. Skhosana:** Conceptualization (equal); data curation (lead); formal analysis (lead); investigation (lead); methodology (equal); project administration (lead); resources (lead); visualization (lead); writing – original draft (lead); writing – review and editing (equal). **Guy F. Midgley:** Conceptualization (equal); investigation (supporting); methodology (equal); resources (supporting); supervision (lead); validation (supporting); writing – review and editing (equal). **Graham von Maltitz:** Conceptualization (equal); funding acquisition (lead); investigation (supporting); methodology (equal); resources (supporting); supervision (supporting); validation (supporting); writing – review and editing (equal). **Mohau J. Mateyisi:** Conceptualization (supporting); formal analysis (supporting); investigation (supporting); methodology (supporting); resources (supporting); supervision (supporting); validation (supporting); visualization (supporting); writing – review and editing (equal). **Humbelani F. Thenga:** Data curation (supporting); investigation (supporting); writing – review and editing (supporting). **Nicola Stevens:** Conceptualization (equal); data curation (supporting); formal analysis (supporting); investigation (supporting); methodology (equal); resources (supporting); supervision (equal); validation (supporting); visualization (supporting); writing – review and editing (equal).

## ACKNOWLEDGMENTS

We extend our gratitude to Mr. Happy Mangena for assisting with data collection. We also thank Prof. Wayne Twine for his support during data collection at Wits Rural Facility.

## FUNDING INFORMATION

The study was funded by the SA‐ICON Project (P1CGC00) under a PhD studentship and the Seamless Forecasting System Capability Development Project (P1DCM00) within the Council for Scientific and Industrial Research (CSIR). We also thank the University of Stellenbosch for supporting the study.

## CONFLICT OF INTEREST STATEMENT

The authors declare no competing interests.

## Supporting information


Appendix S1

## Data Availability

The rain partitioning experimental data can be found in the Dryad Digital Repository (DOI: https://doi.org/10.5061/dryad.mw6m9061s).

## References

[ece39868-bib-0001] Al‐Ansari, N. , Abdellatif, M. , Ezeelden, M. , Ali, S. S. , & Knutsson, S. (2014). Climate change and future long‐term trends of rainfall at north‐east of Iraq. Journal of Civil Engineering and Architecture, 8(6), 790–805.

[ece39868-bib-0002] Andersen, E. M. , & Steidl, R. J. (2019). Woody plant encroachment restructures bird communities in semiarid grasslands. Biological Conservation, 240, 108276. 10.1016/J.BIOCON.2019.108276

[ece39868-bib-0003] Archer, S. R. (2010). Rangeland conservation and shrub encroachment: New perspectives on an old problem. In J. T. du Toit , R. Kock , & J. C. Deutsch (Eds.), Wild rangelands: Conserving wildlife while maintaining livestock in semi‐arid ecosystems (1st ed.). Blackwell Publishing. 10.1002/9781444317091.ch4

[ece39868-bib-0004] Archibald, S. , & Bond, W. J. (2003). Growing tall vs growing wide: Tree architecture and allometry of Acacia karroo in forest, savanna, and arid environments. Oikos, 102(1), 3–14. 10.1034/j.1600-0706.2003.12181.x

[ece39868-bib-0005] Barthiban, S. , Lloyd, B. J. , & Maier, M. (2012). Sanitary hazards and microbial quality of open dug Wells in the Maldives Islands. Journal of Water Resource and Protection, 4(7), 474–486. 10.4236/JWARP.2012.47055

[ece39868-bib-0006] Bastin, J. F. , Finegold, Y. , Garcia, C. , Mollicone, D. , Rezende, M. , Routh, D. , Zohner, C. M. , & Crowther, T. W. (2019). The global tree restoration potential. Science, 364(6448), 76–79. 10.1126/SCIENCE.AAX0848/SUPPL_FILE/AAX0848_BASTIN_SM_DATA-FILE-S2.CSV 31273120

[ece39868-bib-0007] Belay, T. A. , & Moe, S. R. (2015). Assessing the effects of Woody Plant traits on understory herbaceous cover in a semiarid rangeland. Environmental Management, 56(1), 165–175. 10.1007/S00267-015-0491-3 25860596

[ece39868-bib-0008] Ben‐Shahar, R. (1992). The effects of bush clearance on African ungulates in a semi‐arid nature reserve. Ecological Applications, 2(1), 95–101. 27759193 10.2307/1941892

[ece39868-bib-0009] Bond, W. J. , Stevens, N. , Midgley, G. F. , & Lehmann, C. E. R. (2019). The trouble with trees: Afforestation plans for Africa. Trends in Ecology & Evolution, 34(11), 963–965. 10.1016/J.TREE.2019.08.003 31515117

[ece39868-bib-0010] Carlyle‐Moses, D. (2004). Throughfall, stemflow, and canopy interception loss fluxes in a semi‐arid Sierra Madre oriental matorral community. Journal of Arid Environments, 58, 181–202. 10.1016/S0140-1963(03)00125-3

[ece39868-bib-0011] Carlyle‐Moses, D. E. , & Gash, J. H. C. (2011). Rainfall interception loss by forest canopies. In D. F. Levia , D. E. Carlyle‐Moses , & T. Tanaka (Eds.), Forest hydrology and biogeochemistry: Synthesis of past research and future directions (pp. 407–423). Springer. 10.1007/978-94-007-1363-5_20

[ece39868-bib-0012] Case, M. F. , Wigley‐Coetsee, C. , Nzima, N. , Scogings, P. F. , & Staver, A. C. (2019). Severe drought limits trees in a semi‐arid savanna. Ecology, 100(11), e02842. 10.1002/ECY.2842 31339179

[ece39868-bib-0013] Charles‐Dominique, T. , Midgley, G. F. , Tomlinson, K. W. , & Bond, W. J. (2018). Steal the light: Shade vs fire adapted vegetation in forest – Savanna mosaics. New Phytologist, 218, 1419–1429. 10.1111/nph.15117 29604213

[ece39868-bib-0014] Crockford, R. H. , & Richardson, D. P. (2000). Partitioning of rainfall into throughfall, stemflow and interception: Effect of forest type, ground cover and climate. Hydrological Processes, 14, 2903–2920. 10.1002/1099-1085

[ece39868-bib-0015] de Klerk, J. N. (2004). Bush encroachment in Namibia. Report on phase 1 of the bush encroachment research, monitoring and management project. In Ministry of environment and tourism. Government of the Republic of Namibia.

[ece39868-bib-0016] Dohnal, M. , Černý, T. , Votrubová, J. , & Tesař, M. (2014). Rainfall interception and spatial variability of throughfall in spruce stand. Journal of Hydrology and Hydromechanics, 62(4), 277–284. 10.2478/johh-2014-0037

[ece39868-bib-0017] Eldridge, D. J. , Bowker, M. A. , Maestre, F. T. , Roger, E. , Reynolds, J. F. , & Whitford, W. G. (2011). Impacts of shrub encroachment on ecosystem structure and functioning: Towards a global synthesis. Ecology Letters, 14(7), 709–722. 10.1111/j.1461-0248.2011.01630.x 21592276 PMC3563963

[ece39868-bib-0018] Falster, D. S. , & Westoby, M. (2003). Leaf size and angle vary widely across species: What consequences for light interception? New Phytologist, 158(3), 509–525. 10.1046/J.1469-8137.2003.00765.X 36056508

[ece39868-bib-0019] Grygoruk, M. , Batelaan, O. , Mirosław‐Swia˛tek, D. , Szatyłowicz, J. , & Okruszko, T. (2014). Evapotranspiration of bush encroachments on a temperate mire meadow – A nonlinear function of landscape composition and groundwater flow. Ecological Engineering, 73, 598–609. 10.1016/j.ecoleng.2014.09.041

[ece39868-bib-0020] Honda, E. A. , & Durigan, G. (2016). Woody encroachment and its consequences on hydrological processes in the savannah. Philosophical Transactions of the Royal Society B: Biological Sciences, 371(1703), 20150313. 10.1098/rstb.2015.0313 PMC4978871 27502378

[ece39868-bib-0101] Kaschula, S. A. , Twine, W. E. , & Scholes, M. C. (2005). Coppice harvesting of fuelwood species on a South African common: Utilizing scientific and indigenous knowledge in community based natural resource management. Human Ecology, 33(3), 387–418. 10.1007/s10745-005-4144-7

[ece39868-bib-0022] Khan, M. A. (1999). Water balance and hydrochemistry of precipitation components in forested ecosystems in the arid zone of Rajasthan, India. Hydrological Sciences Journal, 44(2), 149–161. 10.1080/02626669909492214

[ece39868-bib-0023] Klein Tank, A. M. G. , Zwiers, F. W. , & Zhang, X. (2009). Guidelines on analysis of extremes in a changing climate in support of informed decisions for adaptation . http://www.clivar.org/organization/etccdi/etccdi.php

[ece39868-bib-0024] Krämer, I. , & Hölscher, D. (2009). Rainfall partitioning along a tree diversity gradient in a deciduous old‐growth forest in Central Germany. Ecohydrology, 2(1), 102–114. 10.1002/eco.44

[ece39868-bib-0025] Levia, D. F. , & Frost, E. E. (2003). A review and evaluation of stemflow literature in the hydrologic and biogeochemical cycles of forested and agricultural ecosystems. Journal of Hydrology, 274, 1–29. 10.1016/S0022-1694(02)00399-2

[ece39868-bib-0102] Li, X. Y. , Liu, L. Y. , Gao, S. Y. , Ma, Y. J. , & Yang, Z. P. (2008). Stemflow in three shrubs and its effect on soil water enhancement in semiarid loess region of China. Agricultural and Forest Meteorology, 148(10), 1501–1507. 10.1016/j.agrformet.2008.05.003

[ece39868-bib-0026] Li, X. , Xiao, Q. , Niu, J. , Dymond, S. , van Doorn, N. S. , Yu, X. , Xie, B. , Lv, X. , Zhang, K. , & Li, J. (2016). Process‐based rainfall interception by small trees in northern China: The effect of rainfall traits and crown structure characteristics. Agricultural and Forest Meteorology, 218–219, 65–73. 10.1016/j.agrformet.2015.11.017

[ece39868-bib-0027] Magliano, P. N. , Whitworth‐Hulse, J. I. , & Baldi, G. (2019). Interception, throughfall and stemflow partition in drylands: Global synthesis and meta‐analysis. Journal of Hydrology, 568, 638–645. 10.1016/J.JHYDROL.2018.10.042

[ece39868-bib-0028] Martens, S. N. , Breshears, D. D. , & Meyer, C. W. (2000). Spatial distributions of understory light along the grassland/forest continuum: Effects of cover, height, and spatial pattern of tree canopies. Ecological Modelling, 126(1), 79–93. 10.1016/S0304-3800(99)00188-X

[ece39868-bib-0103] Martinez‐Meza, E. , & Whitford, W. G. (1996). Stemflow, throughfall and channelization of stemflow by roots in three Chihuahuan desert shrubs. Journal of Arid Environments, 32(3), 271–287. 10.1006/JARE.1996.0023

[ece39868-bib-0029] Meik, J. M. , Jeo, R. M. , Mendelson, J. R. , & Jenks, K. E. (2002). Effects of bush encroachment on an assemblage of diurnal lizard species in Central Namibia. Biological Conservation, 106(1), 29–36. 10.1016/S0006-3207(01)00226-9

[ece39868-bib-0030] Milly, P. C. D. , Dunne, K. A. , & Vecchia, A. V. (2005). Global pattern of trends in streamflow and water availability in a changing climate. Nature, 438(7066), 347–350. 10.1038/nature04312 16292308

[ece39868-bib-0031] Moncrieff, G. R. , Lehmann, C. E. R. , Schnitzler, J. , Gambiza, J. , Hiernaux, P. , Ryan, C. M. , Shackleton, C. M. , Williams, R. J. , & Higgins, S. I. (2014). Contrasting architecture of key African and Australian savanna tree taxa drives intercontinental structural divergence. Global Ecology and Biogeography, 23(11), 1235–1244. 10.1111/GEB.12205

[ece39868-bib-0104] Moyo, H. (2013). The coppicing of a Savanna tree species (Terminalia Sericea) in relation to resource manipulation and disturbance. University of the Witwatersrand.

[ece39868-bib-0032] Návar, J. , Carlyle‐Moses, D. E. , & Alfonso Martinez, M. (1999). Interception loss from the *Tamaulipan matorral* thornscrub of North‐Eastern Mexico: An application of the Gash analytical interception loss model. Journal of Arid Environments, 41(1), 1–10. 10.1006/JARE.1998.0460

[ece39868-bib-0105] Neke, K. S. (2005). The regeneration ecology of Savanna woodlands in relation to human utilisation. University of the Witwatersrand.

[ece39868-bib-0106] Neke, K. S. , Owen‐Smith, N. , & Witkowski, E. T. F. (2006). Comparative resprouting response of Savanna woody plant species following harvesting: The value of persistence. Forest Ecology and Management, 232, 114–123. 10.1016/j.foreco.2006.05.051

[ece39868-bib-0033] Osborne, C. P. , Charles‐Dominique, T. , Stevens, N. , Bond, W. J. , Midgley, G. , & Lehmann, C. E. R. (2018). Human impacts in African savannas are mediated by plant functional traits. New Phytologist, 220(1), 10–24. 10.1111/nph.15236 29806964

[ece39868-bib-0034] Owens, M. K. , Lyons, R. K. , & Alejandro, C. L. (2006). Rainfall partitioning within semiarid juniper communities: Effects of event size and canopy cover. Hydrological Processes, 20, 3179–3189. 10.1002/hyp.6326

[ece39868-bib-0035] Pérez‐Suárez, M. , Arredondo‐Moreno, J. T. , Huber‐Sannwald, E. , & Serna‐Pérez, A. (2014). Forest structure, species traits and rain characteristics influences on horizontal and vertical rainfall partitioning in a semiarid pine‐oak forest from Central Mexico. Ecohydrology, 7(2), 532–543. 10.1002/ECO.1372

[ece39868-bib-0036] Pflug, S. , Voortman, B. R. , Cornelissen, J. H. C. , & Witte, J. P. M. (2021). The effect of plant size and branch traits on rainfall interception of 10 temperate tree species. Ecohydrology, 14(8), e2349. 10.1002/ECO.2349

[ece39868-bib-0037] Pilgrim, D. H. , Chapman, T. G. , & Doran, D. G. (1988). Problems of rainfall‐runoff modelling in arid and semiarid regions. Hydrological Sciences Journal, 33(4), 379–400. 10.1080/02626668809491261

[ece39868-bib-0038] Randle, M. , Stevens, N. , & Midgley, G. (2018). Comparing the differential effects of canopy shading by *Dichrostachys cinerea* and *Terminalia sericea* on grass biomass. South African Journal of Botany, 119, 271–277. 10.1016/j.sajb.2018.09.026

[ece39868-bib-0039] Ratnam, J. , Bond, W. J. , Fensham, R. J. , Hoffmann, W. A. , Archibald, S. , Lehmann, C. E. R. , Anderson, M. T. , Higgins, S. I. , & Sankaran, M. (2011). When is a ‘forest’ a savanna, and why does it matter? Global Ecology and Biogeography, 20(5), 653–660. 10.1111/J.1466-8238.2010.00634.X

[ece39868-bib-0040] Rigby, R. A. , & Stasinopoulos, D. M. (2005). Generalized additive models for location, scale and shape. Journal of the Royal Statistical Society. Series C Applied Statistics, 54(3), 507–554. 10.1111/J.1467-9876.2005.00510.X

[ece39868-bib-0107] Roques, K. G. , O’Connor, T. G. , & Watkinson, A. R. (2001). Dynamics of shrub encroachment in an African savanna: Relative influences of fire, herbivory, rainfall and density dependence. Journal of Applied Ecology, 38(2), 268–280. 10.1046/j.1365-2664.2001.00567.x

[ece39868-bib-0041] Roth, B. E. , Slatton, C. K. , & Cohen, M. J. (2007). On the potential for high‐resolution lidar to improve rainfall interception estimates in forest ecosystems. Frontiers in Ecology and the Environment, 5(8), 421–428. 10.1890/060119.01

[ece39868-bib-0108] Rutter, A. J. (1963). Studies in the water relations of *Pinus sylvestris* in plantation conditions I. Measurements of rainfall and interception. Journal of Ecology, 51(1), 191–203.

[ece39868-bib-0042] Savenije, H. H. G. (2004). The importance of interception and why we should delete the term evapotranspiration from our vocabulary. Hydrological Processes, 18(8), 1507–1511. 10.1002/hyp.5563

[ece39868-bib-0043] Scholes, R. J. , & Archer, S. R. (1997). Tree‐grass interactions in savannas. Annual Review of Ecology and Systematics, 28(1), 517–544. 10.1146/annurev.ecolsys.28.1.517

[ece39868-bib-0044] Scott, R. L. , Huxman, T. E. , Williams, D. G. , & Goodrich, D. C. (2006). Ecohydrological impacts of woody‐plant encroachment: Seasonal patterns of water and carbon dioxide exchange within a semiarid riparian environment. Global Change Biology, 12(2), 311–324. 10.1111/J.1365-2486.2005.01093.X

[ece39868-bib-0109] Shackleton, C. M. (1993). Fuelwood harvesting and sustainable utilisation in a communal grazing land and protected area of the eastern transvaal lowveld. Biological Conservation, 63(3), 247–254. 10.1016/0006-3207(93)90720-L

[ece39868-bib-0110] Shackleton, C. M. (2001). Managing regrowth of an indigenous savanna tree species (*Terminalia sericea*) for fuelwood: The influence of stump dimensions and post‐harvest coppice pruning. Biomass and Bioenergy, 20, 261–270. 10.1016/S0961-9534(00)00086-6

[ece39868-bib-0045] Stevens, N. , Lehmann, C. E. R. , Murphy, B. P. , & Durigan, G. (2017). Savanna woody encroachment is widespread across three continents. Global Change Biology, 23(1), 235–244. 10.1111/gcb.13409 27371937

[ece39868-bib-0046] Tear, T. H. , Wolff, N. H. , Lipsett‐Moore, G. J. , Ritchie, M. E. , Ribeiro, N. S. , Petracca, L. S. , Lindsey, P. A. , Hunter, L. , Loveridge, A. J. , & Steinbruch, F. (2021). Savanna fire management can generate enough carbon revenue to help restore Africa's rangelands and fill protected area funding gaps. One Earth, 4(12), 1776–1791. 10.1016/J.ONEEAR.2021.11.013

[ece39868-bib-0047] Venter, Z. , Cramer, M. D. , & Hawkins, H. (2018). Drivers of woody plant encroachment over Africa. Nature Communications, 2018, 2272. 10.1038/s41467-018-04616-8 PMC5995890 29891933

[ece39868-bib-0048] Wagenmakers, E.‐J. , Farrell, S. , & Wagenmakers, J. (2004). AIC model selection using Akaike weights. Psychonomic Bulletin & Review, 11, 192–196. 15117008 10.3758/bf03206482

[ece39868-bib-0049] Wang, J. , Xiao, X. , Zhang, Y. , Qin, Y. , Doughty, R. B. , Wu, X. , Bajgain, R. , & Du, L. (2018). Enhanced gross primary production and evapotranspiration in juniper‐encroached grasslands. Global Change Biology, 24(12), 5655–5667. 10.1111/GCB.14441 30215879

[ece39868-bib-0050] Wang, L. , D'Odorico, P. , O'Halloran, L. R. , Caylor, K. , & Macko, S. (2009). Combined effects of soil moisture and nitrogen availability variations on grass productivity in African savannas. Plant and Soil, 328, 95–108. 10.1007/S11104-009-0085-Z

[ece39868-bib-0051] Zhang, Y. F. , Feng, Y. , Wang, X. P. , Hu, R. , Pan, Y. X. , & Paradeloc, M. (2015). Rainfall partitioning into throughfall, stemflow and interception loss by two xerophytic shrubs within a rain‐fed re‐vegetated desert ecosystem, northwestern China. Journal of Hydrology, 527, 1084–1095. 10.1016/j.jhydrol.2015.05.060

[ece39868-bib-0052] Zhang, Y. , Li, X.‐Y. , Li, W. , Wu, X.‐C. , Shi, F.‐Z. , Fang, W.‐W. , & Pei, T.‐T. (2017). Modeling rainfall interception loss by two xerophytic shrubs in the loess plateau. Hydrological Processes, 31(10), 1926–1937. 10.1002/HYP.11157

[ece39868-bib-0053] Zizka, A. , Govender, N. , & Higgins, S. I. (2014). How to tell a shrub from a tree: A life‐history perspective from a south African savanna. Austral Ecology, 39(7), 767–778. 10.1111/AEC.12142

