# Supplementary material for: Steal the rain: Interception loses and rainfall partitioning by a broad‐leaf and a fine‐leaf woody encroaching species in a southern African semi‐arid savanna
Source: Ecol Evol. 2023 Mar 15;13(3):e9868. doi: 10.1002/ece3.9868 (PMC10017313; doi:10.1002/ece3.9868)
Supplement: Supplementary file 1 — Appendix S1 [file ECE3-13-e9868-s001.pdf]

Characteristics of woody encroachment

Dense stands (highly encroached) of *D. cinerea* were characterized by multiple thin stems (mean number of 20 stems per plot, with a mean stem diameter of 4.1 cm). Dense stands (highly encroached) of *T. sericea* encroachment were characterized by few, thick stems (17 stems per plot with mean diameter of 6.9 cm) (Table S1). On average *D. cinerea* plots had a high canopy cover (LAI) of 2.2 compared to 1.5 in *T. sericea* (Table S1).

Table S1: Woody cover characteristics of the two quantified species at plot level

**Woody cover characteristics at**	***D.cinerea***			***T.sericea***		
**plot level**	Low	Medium	High	Low	Medium	High
**Mean height (m)**	4.9	4.0	4.6	5.5	5.4	5.6
**Mean stem density**	5	21	43	1	6	17
**Mean stem diameter (cm)**	4.8	4.5	4.1	13.5	8.5	6.9
**Leaf Area Index (LAI)**	0.8	1.9	2.22	0.9	1.0	1.5
**TBA (m^2/ha)**	4.3	13.1	23.8	7.3	15.1	26.7

The tree basal area (TBA) positively correlated ($r^2 > 0.5$) with stem density and LAI (Figure 3). To maintain consistency with other previous similar studies (such as Honda and Durigan 2016), TBA was chosen as a proxy for woody cover among the three measures.

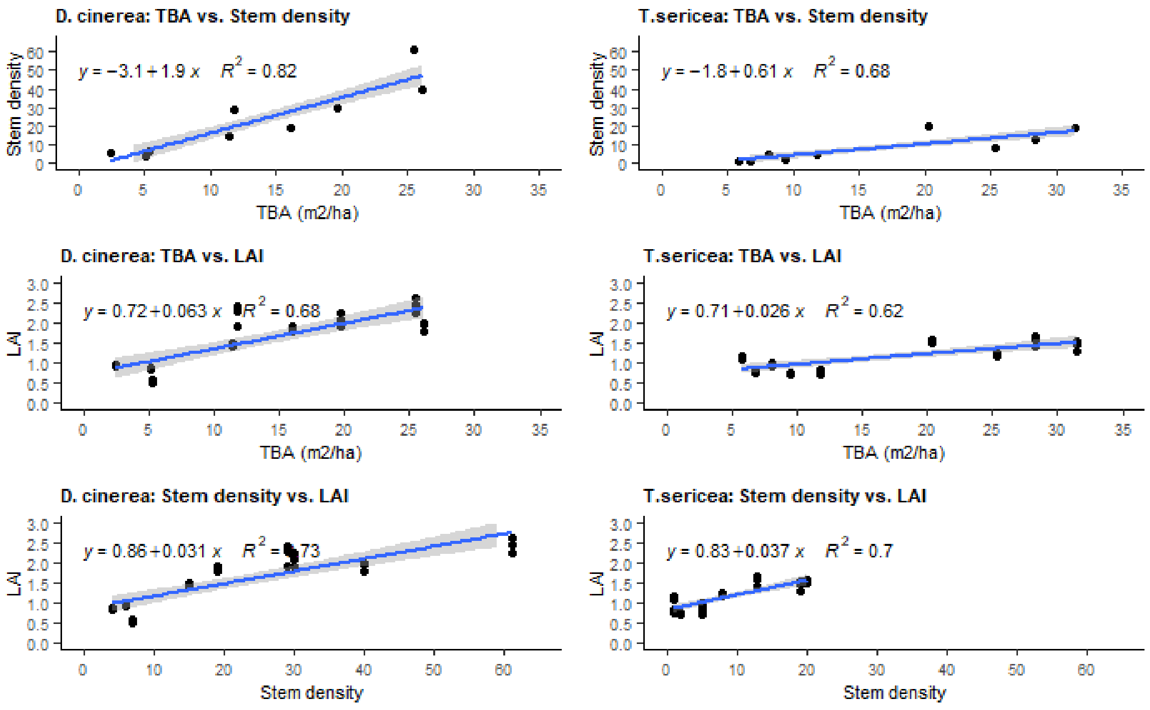

Figure S1: Correlations between (a) tree basal area (TBA) and stem density, and (b) TBA and leaf area index (LAI). Each point on the scatter plot represents a mean value at plot level. Grey bands are the 95% confidence intervals.

Model distributions and outputs

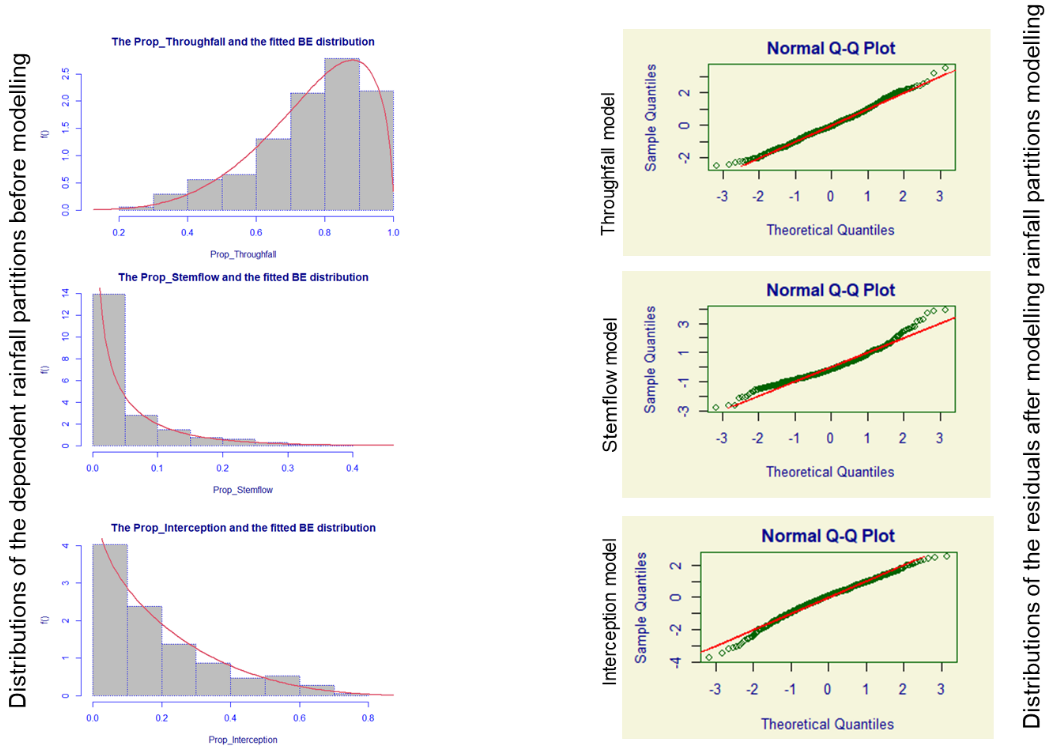

Figure S2: Histograms of the beta distribution of the proportions of throughfall, stemflow and interception before modeling the data and the Q-Q plots of the residuals of the fitted beta regression models for predicting throughfall, stemflow and interception in response to woody encroachment and rainfall characteristics.

Table S2: generalized mixed effects model outputs for the three partitions of rainfall in response to tree basal area, species, rainfall size and rainfall intensity.

	Prop_Throughfall				Prop_Stemflow				Prop_Interception			
Predictors	*Estimates*	*std. Error*	*CI*	*p*	*Estimates*	*std. Error*	*CI*	*p*	*Estimates*	*std. Error*	*CI*	*p*
(Intercept)	1.52	0.09	1.35 – 1.69	**<0.001**	-4.76	0.12	-5.00 – -4.52	**<0.001**	-1.45	0.10	-1.65 – -1.24	**<0.001**
Plot TBA m2 ha	-0.05	0.00	-0.06 – -0.04	**<0.001**	0.05	0.01	0.04 – 0.07	**<0.001**	0.04	0.01	0.03 – 0.05	**<0.001**
Species [T.sericea]	0.21	0.11	-0.01 – 0.42	0.067	0.12	0.13	-0.13 – 0.36	0.364	-0.25	0.13	-0.52 – 0.01	0.056
Rain_class_mm(10-20mm]	0.48	0.15	0.19 – 0.78	**0.001**	1.13	0.16	0.82 – 1.44	**<0.001**	-0.63	0.18	-0.99 – -0.28	**0.001**
Rain class mm [>20mm]	0.50	0.21	0.10 – 0.91	**0.015**	1.21	0.21	0.80 – 1.62	**<0.001**	-0.80	0.25	-1.29 – -0.31	**0.002**
Rain intensity class [Moderate rain (2.5 - 7.5 mm/hr]]	0.15	0.15	-0.14 – 0.45	0.302	0.35	0.17	0.03 – 0.68	**0.033**	-0.10	0.18	-0.45 – 0.26	0.594
Plot TBA m2 ha * Species [T.sericea]	0.02	0.01	0.01 – 0.03	**<0.001**	-0.02	0.01	-0.03 – -0.01	**0.003**	-0.02	0.01	-0.03 – -0.00	**0.038**
Plot_TBA_m2.ha:Rain_class_mm(10-20mm]	-0.01	0.01	-0.03 – 0.00	0.136	0.04	0.01	0.02 – 0.05	**<0.001**	-0.02	0.01	-0.04 – 0.00	0.061
Plot TBA m2 ha * Rain class mm [>20mm]	-0.01	0.01	-0.03 – 0.01	0.252	0.02	0.01	-0.01 – 0.04	0.153	-0.00	0.01	-0.03 – 0.03	0.943
Plot TBA m2 ha * Rain intensity class [Moderate rain (2.5 - 7.5 mm/hr]]	0.01	0.01	-0.01 – 0.03	0.210	-0.00	0.01	-0.02 – 0.02	0.901	-0.02	0.01	-0.04 – -0.00	**0.029**
(Intercept)	-0.87	0.04	-0.94 – -0.80	**<0.001**	-1.50	0.04	-1.57 – -1.42	**<0.001**	-0.60	0.04	-0.68 – -0.53	**<0.001**
N_{Site1}	3				3				3			
Observations	620				620				620			
